# Early Dietary Patterns and Microbiota Development: Still a Way to Go from Descriptive Interactions to Health-Relevant Solutions

**DOI:** 10.3389/fnut.2018.00005

**Published:** 2018-02-02

**Authors:** Patricia Iozzo, Elena Sanguinetti

**Affiliations:** ^1^Institute of Clinical Physiology, National Research Council, Pisa, Italy

**Keywords:** milk, formula, obesity, children, probiotic, prebiotic, maternal, complementary food

## Abstract

Early nutrition and growth in the initial years of life are important determinants of later body weight and metabolic health in humans, and the current epidemic of obesity involving children requires a better understanding of causal and protective mechanisms and components in infant foods. This review focuses on recent evidence implicating feeding modes (e.g., breast milk and formula milk) and dietary transitions toward complementary foods in the progression of microbiota maturation in children. The literature exploring body weight outcomes of microbiota changes induced by diet in early life is limited. Representative studies addressing the use of probiotics in pregnant women and infants are also examined. Methodological and geo-cultural variations make it difficult to avoid (apparently) controversial findings. Most studies indicate differences in the microbiota of formula versus breastfed infants, but some do not. Duration of breastfeeding delays the maturation of the microbiota toward an adult-like profile. However, the effect size of the early feeding pattern on microbial function was found to be very small, and absent after the third year of life. There are several interesting mediators whereby milk composition can affect infants’ microbiota and their optimization is a desirable strategy for prevention. But prevention of what? Although there are few correlative evaluations relating microbiota and body weight in early life, studies demonstrating a cause–effect relationship between diet-induced changes in early microbiota development and subsequent metabolic health outcomes in humans are still missing.

## Introduction

Associations between microbiota composition and obesity in humans have been repeatedly confirmed, although the cumulating effects of diseases, medications, genetic, and environmental factors make it difficult to dissect causative from adaptive microbial changes in adults. Life course studies would help to identify primary and secondary events and critical time windows. The transition from birth to adulthood results in profound modifications of the microbiota, which is characterized by a dominance of Firmicutes and Bacteroidetes in adults, as opposed to infants, in whom Actinobacteria and Firmicutes are dominant, Proteobacteria are more represented, and Bacteroidetes are nearly or entirely absent, especially in newborns (1, 2). Early nutrition predicts the later obesity risk and drives microbiota development. Considering the relevance of early life in gut colonization and microbiota stabilization, it is plausible to hypothesize that early life dysbiosis could be prevented or corrected by optimizing nutritional patterns. This minireview summarizes studies examining the longitudinal development of the gut microbiota from infancy to early childhood in relation to breastfeeding, complementary foods, probiotic interventions, and the potential link with childhood obesity.

## Microbiota Development Over Feeding Transitions

There are few longitudinal studies covering the period from birth to infancy or early childhood. In a Swedish cohort (3) of 98 mothers undergoing gut microbiota analysis at 2 days of delivery, and their full-term infants studied in the first week of life, at 4 and 12 months of age, a 72% correspondence in MetaOTUs in mother–newborn pairs was observed after vaginal delivery, compared with 41% after C-section, indicative of vertical transmission. In spontaneously delivered newborns, the gut was initially colonized by relatively aerobic bacteria (*Enterococcus, Escherichia/Shigella, Streptococcus*, and *Rothia*), followed during lactation by anaerobic bacteria involved in lactate metabolism (*Bifidobacterium, Lactobacillus, Collinsella, Granulicatella*, and *Veillonella*), and in complementary feeding period by bacteria involved in fiber and carbohydrates degradation, and short-chain fatty acid production (*Bacteroides, Bilophila, Roseburia, Clostridium*, and *Anaerostipes*), resembling maternal microbiota. Consistently, the repeated characterization of 192 breastfed infants (4) from 1 week to 6 months of age to primarily addresses the effects of gestational age and delivery mode, showed a progressive reduction in genera that are abundant at 1 week toward genera that become abundant at subsequent ages, including (from 8 weeks) those that are core to enterotypes, such as *Prevotella, Blautia*, and *Ruminococcus*. On a functional level (3), genes for carbohydrate uptake, vitamin metabolism, and essential amino acid transport were enriched in newborns microbiome, while functions involved in lactose transport were most abundant in 4-month-old infants, and those implicated in degradation of complex sugars, starch, pectin, and in methionine degradation, lysine biosynthesis, and carbohydrate, leucine, and tryptophan metabolism, were enriched at 12 months. The subsequent period of life was examined in 264–311 Danish children (5) studied at 9, 18, and 36 months, showing that microbial composition at 9 months maintains an influence of the preceding milk-based diet, whereas dramatic changes occur between 9 and 18 months, followed by fewer adaptations later. This suggested that the period necessary for microbiome stabilization is longer than previously claimed. The progression from 9 to 36 months was primarily characterized by a reduction in *Bifidobacterium* (*longum* and *breve*), *Lactobacillus* spp., *Enterobacteriaceae, Clostridium coccoides*, and by an increase in Bacteroidetes-related species, *Clostridium leptum, Eubacterium hallii, Roseburia* spp., and *Bifidobacterium* (*adolescentis* and *catenulatum*). In addition, the initial establishment of human enterotypes, expressed as *Prevotella*/*Bacteroides* ratio, was observed between 18 and 36 months of age, reflecting the transition toward an adult-like microbiota, adapting to an adult-type diet.

Overall, despite a large number of intervening factors, nutrients seem to be dominant regulators of gut microbiota development across the first 3 years of life.

## Microbiota Development and Breast or Formula Feeding in Early Infancy

In the study by Bäckhed et al. (3), 70% of infants were exclusively breastfed at the age of 4 months, showing increased levels of *Lactobacillus* (*johnsonii/gasseri* and *paracasei/casei*), and *Bifidobacterium longum*, compared with formula-fed infants, in whom *Clostridium difficile, Granulicatella adiacens, Citrobacter* spp., *Enterobacter cloacae, Bilophila wadsworthia*, and *Bifidobacterium adolescentis* were more dominant. At a functional level, formula feeding was associated with enrichment in functions typical of the adult microbiota, whereas exclusive breastfeeding was characterized by functions of oxidative phosphorylation and vitamin B synthesis (3). However, the feeding pattern explained only 1.3% of the functional variation. In a Finnish study, a higher number of IgG-secreting cells was observed in 3-month-old infants undergoing exclusive compared with non-exclusive breastfeeding (6), suggesting functional immune-related effects.

The above findings on microbiota composition were partly confirmed or extended by studies in Swedish infants aged 6 days, 3 weeks, 2 and 6 months (1), in Finnish 3, 6, and 12-month-old (6), in Canadian 4-month-old (2), and in North American 3-month-old infants (7), showing that formula-fed infants had increased richness of species, with overrepresentation of *C. difficile* (1, 7), whereas breastfed infants had higher relative abundance of *Bacteroides* (7) and *Bifidobacterium* (6), and lower abundance of *Clostridium, Peptostreptococcaceae*, and *Verrucomicrobiaceae*, including *Akkermansia* genus (2), *Lachnospiraceae incertae sedis, Streptococcus, Enterococcus*, and *Veillonella* (7). Results from other studies (4, 7, 8) are partially at variance with these findings. In a UK cohort (4), infants were stratified in subgroups receiving breastfeeding until 2, 4, and beyond 4 months (feces at 1, 4, 8, and 24 weeks), observing no influence of the feeding pattern on microbiota composition, with the exception of 24-week-old infants delivered by C-section, in whom the abundance of five genera was enhanced by breastfeeding protraction beyond 4 months, suggesting that breastfeeding may compensate for the C-section-dependent deficiency in selected bacteria. Unexpectedly, *Bifidobacterium* abundance did not differ in any groups. Likewise, in a French study (8), absolute bacteria and *Bifidobacterium* abundances were measured on feces collected during 3 subsequent periods in which 11 infants received exclusive breastfeeding (2–11 weeks), mixed feeding (6–21 weeks), and exclusive formula feeding (7–42 weeks), showing no difference across them, due to formula–milk integration or breast-milk exclusion. These two studies do not exclude a long-term impact of early breastfeeding on microbiota development.

Different methodologies, types of formula used, and cohort characteristics (e.g., inclusion of preterm infants, differing frequencies of delivery modes, and antibiotic use), depending on the focus of investigation, might explain some discrepancies between studies. Furthermore, the use of relative or absolute bacterial counts may impact physiological interpretations.

In summary, there is evidence that in the first few months of life, breastfeeding favors the development of health-promoting microbes, whereas formulas stimulate growth of disease-related bacteria, but there are also studies showing marginal or no effects of such dietary patterns on microbiota. Even when a strong impact of early diet on microbiota composition was found, microbial functional properties seemed to be marginally affected (3), challenging the mechanisms that may link microbiota functions and health.

## Microbiota Maturation and Duration of Breastfeeding

One relevant question to understand mechanisms relating milk–microbiota interactions with health outcomes is whether the duration of breastfeeding has long-lasting impact on microbiota development. In 227 Danish infants (2 cohorts, 9) characterized at 9 and 18 months of age, duration of exclusive breastfeeding in 9-month olds was positively correlated with the abundance of bacteria that utilize milk-derived oligosaccharides or lactate (*Bifidobacteria, Veillonellaceae*, and *Pasteurellaceae*), and negatively associated with the abundance of species that utilize plant-derived complex carbohydrates and resistant starch from solid foods, i.e., *Lachnospiraceae* (*Dorea, Coprococcus, Blautia, Pseudobutyrivibrio*, and *Roseburia* genera), *Ruminococcaceae* (*Ruminococcus, Anaerotruncus, Oscillibacter, Clostridium* IV, and *Butyricicoccus* genera), *Erysipelotrichaceae, Peptostreptococcaceae*, and *Eubacteriaceae*.

Interestingly, children fed formula since birth underwent premature microbiota maturation (3), whereas continued breastfeeding at 9–12 months delayed this process, with persistence of high relative levels of *Bifidobacterium* (spp. and *longum*), *Lactobacillus, Collinsella, Megasphaera*, and *Veillonella* (5, 9), and low levels of butyrate-producing taxa, such as *Clostridia* (*leptum* and *coccoides*), *E. hallii*, and *Roseburia* spp., and of *Desulfovibrio* spp., *Akkermansia muciniphila*, and Bacteroidetes species. Coherently, daily breast-milk intake at 9 months was quantitatively correlated with microbiota composition (9), and alpha diversity was lower at increasing durations of exclusive breastfeeding.

In summary, the effects of breastfeeding on microbiota composition are protracted to the second semester of life but become limited and vanish at 18 and 36 months of age (5, 9). Selected differences were abolished already at 12 months of age (6). Once confirmed, these findings convey fundamental mechanistic insight: if breastfeeding impacts later health through the microbiota (to be demonstrated), the mediating mechanism in tissues should be settled during the first year of life, and studying the microbiota as a function of breastfeeding in later life may not elucidate that causal link.

## Milk Components Affecting Infant’s Microbiota

One potential source of variability and discrepancy between studies may be the composition of breast milk or formula due to, e.g., geo-cultural differences, maternal factors, industrial production, and distribution. Properties whereby breast milk may influence child’s microbiota establishment include the vertical transmission of bacteria, the oligosaccharides-stimulated bacterial growth, and the exposure to immune modulators. In a UK study (10), 10 mother–infant pairs underwent collection of maternal milk and feces at 1, 3, 6, and 12 weeks of newborns life. The milk microbiota showed high diversity and changes in dominant microbes over time. Intriguingly, milk microbiota composition accounted for the 70–88% of infant’s microbial abundance, and identical strains of *Bifidobacterium breve* and *Lactobacillus plantarum* were observed in maternal milk and respective infant gut. Milk bacteria can originate from maternal skin, neonatal oral cavity, or maternal gut (enteromammary pathway) and are influenced by delivery mode (11), with higher bacterial diversity and richness in response to vaginal compared with C-section delivery. Considering that samples were collected after 1 month of breastfeeding, the influence of neonatal oral bacteria is a possible explanation, since the newborn microbiota (at least in the gut) is by itself influenced by delivery mode.

Breast milk is rich in oligosaccharides. Oligosaccharides are present in formulas, explaining some of the above controversies (7, 12), although the relative prevalence of these sugars is different (13). In a study relating milk oligosaccharides and microbiota of 3-month-old infants (7), 141 oligosaccharides were identified in breast milk. The authors found that each oligosaccharide predicted multiple microbial changes, and each of the affected microbial abundances was predicted by different oligosaccharides. Gut *Bifidobacterium, Bacteroides, Enterococcus, Veillonella*, and *Rothia* were impacted. Corroborating a cause–effect relationship, infants receiving oligosaccharides-supplemented formula (14) showed a fecal *Bifidobacteria* abundances and composition similar to breastfed infants, exceeding those observed in formula-fed infants.

Breast milk is a source of secretory-IgA, leading to antigen-specific gut immune protection. Mice studies (15) indicated that an early gut exposure to maternal secretory IgA modulates microbiota composition and prevents translocation of aerobic bacteria from the neonatal gut into draining lymph nodes. The effect was persistent and greater in adults, in which the expression of genes implicated in inflammatory diseases by intestinal epithelial cells was also modified. Moreover, maternal secretory IgAs were shown to be protective against colonic damage caused by an epithelial-disrupting agent. These findings reveal mechanisms through which breastfeeding and secretory IgA may protect intestinal health. A Finnish study (6) reported positive associations between levels of soluble cluster of differentiation 14 (sCD14) in maternal colostrum and circulating immunoglobulin-secreting cells in 3 (IgG cells) and 12-month-old offspring (IgA and IgM cells), supporting the role of breast milk sCD14 in influencing infants’ immune intestinal and humoral responses.

In summary, the characterization of milk-related effects on the microbiota may hold important preventive implications, considering that both breast milk and formula compositions can be theoretically manipulated. Identifying the desirable health-related microbial profile in infants would make it possible to design interventions that optimize milk quality. To this end, much effort is required to prove cause–effect relationships between early-life bacteria and later health outcomes.

## Breastfeeding–Microbiota Interaction as Cause of Obesity

Longer duration of breastfeeding is dose-dependently associated with a decrease in later overweight risk (16). Consistently, duration of breast-milk consumption was negatively associated with the overall energy intake (5, 17), and breastfed infants were leaner than formula-fed children at 9 and 18 months in Danish cohorts (5, 18). Breast milk promoted *Bifidobacteria* development in that study and might underlie these findings. Indeed, low *Bifidobacterium* and high *Staphylococcus aureus* abundances at 6 and 12 months of age predicted weight and obesity in 7-year-old Spanish children (19). Considering that the effects of breastfeeding versus formula feeding on microbiota are not lasting beyond 1–3 years (5, 6, 9), it is possible that weight differences emerged earlier, as weight gain during the first 6 months of life is particularly predictive of later obesity, or that later-developing bacteria, colonizing the gut during complementary feeding, may have contributed to aggravate childhood obesity, as discussed below.

## Complementary Feeding: Interaction with the Microbiota and Obesity

Longitudinal studies covering the transition of microbiota from lactation toward solid food and adult-like diets are limited. A thorough 7-day food questionnaire was collected in the Danish cohorts, suggesting that the progression of infant diet toward family foods, enriched in meat, milk, cheese, and animal fats was associated with a reduction in *Bifidobacteriaceae* and *Enterococcaceae*, in favor of *Lachnospiraceae* and *Sutterellaceae* abundances (9). However, these cohorts did not address microbiota composition before the ninth month of age, and longer observation periods are required to expand the above knowledge.

There is no clear association between the timing of complementary food introduction and childhood overweight or obesity, but some evidence suggests that its introduction at 4 months or earlier, compared with 4–6 months or longer, may increase the risk of childhood overweight (20, 21). Coherently, age at introduction of complementary foods did not correlate with microbial abundances or alpha diversity at 9 months (9). The composition, rather timing of complementary foods has been more clearly related to later obesity risk (22). In particular, high intakes of energy and dairy protein in infancy could be associated with an increase in body weight and fatness. Consistently, correlative results indicated that changes in the microbiota induced by the progression from early infant to family foods are mostly driven by protein and fiber dietary contents (9). However, the microbial categories that changed due to family food (*Bifidobacteriaceae, Enterococcaceae, Lachnospiraceae*, and *Sutterellaceae*), did not correspond to bacteria that were predictive of weight gain. Indeed, the increase in body mass index between 9 and 36 months (5) was predicted by positive changes in Firmicutes, *C. leptum*, and *E. hallii*, i.e., butyrate-producing groups contributing to energy harvest, and negative changes in *Methanobrevibacter smithii* and *Enterobacteriaceae*. Interestingly, these bacteria are the same predicted by breastfeeding duration in earlier life phases, and not by complementary diet characteristics.

## Probiotics and Microbiota Development

Few interventions have been conducted to examine the effects of probiotics on infant’s microbiota or immunity development. A recent double-blind, randomized, placebo-controlled study (23) was conducted in 106 newborns assigned to standard whey-based formula, containing 10^8^ colony-forming units/g of *Bifidobacteria* (*bifidum, breve*, and *longum*), or to control formula (placebo) in a 12-month intervention, followed by 24 months of follow-up to examine microbiome–metabolome outcomes. The conclusion was that supplementation of *Bifidobacteria* to infant diet can modulate the occurrence of specific bacteria, i.e., *Bacteroides* and *Blautia* spp. and metabolites during early life, with no detectable long-term effects. In a 4-week double-blind, placebo-controlled trial (6), 96 mothers were randomized to receive placebo or 10^10^ colony-forming units of *Lactobacillus rhamnosus GG* before delivery. The treatment was protracted in respective infants until 6 months of age, with follow-up visits at 3, 6, and 12 months. The study, mostly oriented to immune-related aspects, demonstrated a significant interaction between probiotics and breastfeeding in relation to the amounts of immunoglobulin-secreting cells observed in infants’ circulation and suggested that *Lactobacilli* may potentiate the beneficial effects of maternal milk on the offspring’s immunity. Similarly, in a randomized, double-blind, placebo-controlled trial, an oral synbiotic preparation (*L. plantarum* plus fructooligosaccharide) administered for 7 days to 4,556 rural Indian newborns following breastfeeding, showed efficacy on neonatal sepsis prevention, suggesting that host–probiotic interactions enhance local gastrointestinal mucosal and systemic host immunity (24).

## Conclusion

Factors affecting early microbiota are numerous and heterogeneous between studies, depending on their focus. They include delivery mode, gestational age, geographical variations in dietary habits, and antibiotic use. Due to their potentially compensatory or synergistic interaction in modulating microbiota development, it is difficult and not necessarily plausible to statistically correct for them. Geographical provenance of cohorts was underlined in this review, since cultural heterogeneity is a potentially important source of actual heterogeneity. Different methods are used to quantify microbial abundance either in relative or absolute terms, which are not of interchangeable interpretation. One reflects the balance between species, in which potentially favorable may overrule the effects of unfavorable bacteria and *vice versa*; the other informs on the independent load of given taxa. The comparison between sequencing techniques goes beyond the scope of this review. The common use of data filtering, and exclusion of large portions of less abundant or frequent taxa, not always clearly justified, may obscure the value of physiologically relevant species and be source of interpretative heterogeneity between studies.

Notwithstanding the above, a majority of reports support a role of early dietary patterns to regulate microbiota composition. Continued breastfeeding appears to delay the transition toward an adult-like microbiota, whereas formula feeding or shorter breastfeeding accelerate this process. It seems important to account for different species of the same genus (e.g., *Bifidobacterium*), which dominate in different life periods. Some controversies remain on the relevance of breastfeeding versus formula feeding, since not all studies could identify a difference in microbiota, especially in very young infants. Complementary food protein and fiber contents, but not age at introduction, were related to microbiota characteristics. There is convincing evidence that milk composition represents an important modulator of infants’ microbiota and intestinal health, and optimization of this interaction is a promising preventive perspective. Some studies have been carried out using pre- or probiotics to influence the development of infants’ microbiota, but their clinical benefits and safety remain to be clearly established.

The most important gap of knowledge relates to the lack of studies including health outcomes. Most studies are descriptive, and some show correlations with body weight, but no cause–effect mechanisms are actually demonstrated. The evidence that early dietary patterns result in a limited functional effect size in metagenome analysis, and that breastfeeding effects on microbiota are no longer seen after the third year of life, or earlier, prompt for studies demonstrating direct cause–effect relationships linking early dietary–microbiota interactions, and specific taxa, with short- and long-term body organ development and health-related consequences.

## Author Contributions

PI conceived, drafted, and reviewed the manuscript. ES contributed to the critical revision of the manuscript, and with PI approved its final version for publication.

## Conflict of Interest Statement

The authors declare that the research was conducted in the absence of any commercial or financial relationships that could be construed as a potential conflict of interest.

## References

[1] Hesla HM Stenius F Jäderlund L Nelson R Engstrand L Alm J Impact of lifestyle on the gut microbiota of healthy infants and their mothers – the ALADDIN birth cohort. FEMS Microbiol Ecol (2014) 90:791–801. 10.1111/1574-6941.12434 25290507

[2] Azad MB Konya T Maughan H Guttman DS Field CJ Chari RS Gut microbiota of healthy Canadian infants: profiles by mode of delivery and infant diet at 4 months. CMAJ (2013) 185(5):385–94. 10.1503/cmaj.121189 23401405 PMC3602254

[3] Bäckhed F Roswall J Peng Y Feng Q Jia H Kovatcheva-Datchary P Dynamics and stabilization of the human gut microbiome during the first year of life. Cell Host Microbe (2015) 17(5):690–703. 10.1016/j.chom.2015.04.004 25974306

[4] Hill CJ Lynch DB Murphy K Ulaszewska M Jeffery IB O’Shea CA Evolution of gut microbiota composition from birth to 24 weeks in the INFANTMET Cohort. Microbiome (2017) 5(1):4 10.1186/s40168-016-0213-y 28095889 PMC5240274

[5] Bergström A Skov TH Bahl MI Roager HM Christensen LB Ejlerskov KT Establishment of intestinal microbiota during early life: a longitudinal, explorative study of a large cohort of Danish infants. Appl Environ Microbiol (2014) 80(9):2889–900. 10.1128/AEM.00342-14 24584251 PMC3993305

[6] Rinne M Kalliomaki M Arvilommi H Salminen S Isolauri E. Effect of probiotics and breastfeeding on the *Bifidobacterium* and *Lactobacillus*/*Enterococcus* microbiota and humoral immune responses. J Pediatr (2005) 147(2):186–91. 10.1016/j.jpeds.2005.03.053 16126047

[7] Wang M Li M Wu S Lebrilla CB Chapkin RS Ivanov I Fecal microbiota composition of breast-fed infants is correlated with human milk oligosaccharides consumed. J Pediatr Gastroenterol Nutr (2015) 60(6):825–33. 10.1097/MPG.0000000000000752 25651488 PMC4441539

[8] Magne F Hachelaf W Suau A Boudraa G Mangin I Touhami M A longitudinal study of infant faecal microbiota during weaning. FEMS Microbiol Ecol (2006) 58(3):563–71. 10.1111/j.1574-6941.2006.00182.x 17117997

[9] Laursen MF Andersen LB Michaelsen KF Mølgaard C Trolle E Bahl MI Infant gut microbiota development is driven by transition to family foods independent of maternal obesity. mSphere (2016) 1(1):e00069–15. 10.1128/mSphere.00069-15 27303699 PMC4863607

[10] Murphy K Curley D O’Callaghan TF O’Shea CA Dempsey EM O’Toole PW The composition of human milk and infant faecal microbiota over the first three months of life: a pilot study. Sci Rep (2017) 7:40597. 10.1038/srep40597 28094284 PMC5240090

[11] Cabrera-Rubio R Mira-Pascual L Mira A Collado MC. Impact of mode of delivery on the milk microbiota composition of healthy women. J Dev Orig Health Dis (2016) 7(1):54–60. 10.1017/S2040174415001397 26286040

[12] Adlerberth I Wold AE. Establishment of the gut microbiota in Western infants. Acta Paediatr (2009) 98:229–38. 10.1111/j.1651-2227.2008.01060.x 19143664

[13] Haarman M Knol J. Quantitative real-time PCR assays to identify and quantify fecal *Bifidobacterium* species in infants receiving a prebiotic infant formula. Appl Environ Microbiol (2005) 71:2318–24. 10.1128/AEM.71.5.2318-2324.2005 15870317 PMC1087546

[14] Rinne MM Gueimonde M Kalliomäki M Hoppu U Salminen SJ Isolauri E. Similar bifidogenic effects of prebiotic-supplemented partially hydrolyzed infant formula and breastfeeding on infant gut microbiota. FEMS Immunol Med Microbiol (2005) 43(1):59–65. 10.1016/j.femsim.2004.07.005 15607637

[15] Rogier EW Frantz AL Bruno ME Wedlund L Cohen DA Stromberg AJ Secretory antibodies in breast milk promote long-term intestinal homeostasis by regulating the gut microbiota and host gene expression. Proc Natl Acad Sci U S A (2014) 111(8):3074–9. 10.1073/pnas.1315792111 24569806 PMC3939878

[16] Harder T Bergmann R Kallischnigg G Plagemann A. Duration of breastfeeding and risk of overweight: a meta-analysis. Am J Epidemiol (2005) 162(5):397–403. 10.1093/aje/kwi222 16076830

[17] Gondolf UH Tetens I Michaelsen KF Trolle E. Dietary habits of partly breast-fed and completely weaned infants at 9 months of age. Public Health Nutr (2012) 15:578–86. 10.1017/S1368980011003247 22152993

[18] Madsen AL Larnkjaer A Molgaard C Michaelsen KF. IGF-I and IGFBP-3 in healthy 9 month old infants from the SKOT cohort: breastfeeding, diet, and later obesity. Growth Horm IGF Res (2011) 21:199–204. 10.1016/j.ghir.2011.05.003 21624842

[19] Kalliomäki M Collado MC Salminen S Isolauri E. Early differences in fecal microbiota composition in children may predict overweight. Am J Clin Nutr (2008) 87(3):534–8. 18326589 10.1093/ajcn/87.3.534

[20] Pearce J Taylor MA Langley-Evans SC. Timing of the introduction of complementary feeding and risk of childhood obesity: a systematic review. Int J Obes (Lond) (2013) 37(10):1295–306. 10.1038/ijo.2013.99 23736360

[21] Wang J Wu Y Xiong G Chao T Jin Q Liu R Introduction of complementary feeding before 4 months of age increases the risk of childhood overweight or obesity: a meta-analysis of prospective cohort studies. Nutr Res (2016) 36(8):759–70. 10.1016/j.nutres.2016.03.003 27440530

[22] Pearce J Langley-Evans SC. The types of food introduced during complementary feeding and risk of childhood obesity: a systematic review. Int J Obes (Lond) (2013) 37(4):477–85. 10.1038/ijo.2013.8 23399778

[23] Bazanella M Maier TV Clavel T Lagkouvardos I Lucio M Maldonado-Gòmez MX Randomized controlled trial on the impact of early-life intervention with bifidobacteria on the healthy infant fecal microbiota and metabolome. Am J Clin Nutr (2017) 106(5):1274–86. 10.3945/ajcn.117.157529 28877893

[24] Panigrahi P Parida S Nanda NC Satpathy R Pradhan L Chandel DS A randomized synbiotic trial to prevent sepsis among infants in rural India. Nature (2017) 548(7668):407–12. 10.1038/nature23480 28813414

